# miR-485-5p Binding Site SNP rs8752 in *HPGD* Gene Is Associated with Breast Cancer Risk

**DOI:** 10.1371/journal.pone.0102093

**Published:** 2014-07-08

**Authors:** Na He, Hong Zheng, Pei Li, Yanrui Zhao, Wei Zhang, Fengju Song, Kexin Chen

**Affiliations:** 1 Department of Epidemiology and Biostatistics, Key Laboratory of Breast Cancer Prevention and Therapy, Ministry of Education, Key Laboratory of Cancer Prevention and Therapy, Tianjin, National Clinical Research Center of Cancer, Tianjin Medical University Cancer Institute and Hospital, Tianjin, P. R. China; 2 Department of Pathology, the University of Texas MD Anderson Cancer Center, Houston, Texas, United States of America; Dartmouth Medical School, United States of America

## Abstract

**Background:**

Single nucleotide polymorphisms (SNPs) that reside in microRNA target sites may play an important role in breast cancer development and progression. To reveal the association between microRNA target site SNPs and breast cancer risk, we performed a large case-control study in China.

**Methods:**

We performed a two-stage case-control study including 2744 breast cancer cases and 3125 controls. In Stage I, we genotyped 192 SNPs within microRNA binding sites identified from the “Patrocles” database using custom Illumina GoldenGate VeraCode assays on the Illumina BeadXpress platform. In Stage II, genotyping was performed on SNPs potentially associated with breast cancer risk using the TaqMan platform in an independent replication set.

**Results:**

In stage I, 15 SNPs were identified to be significantly associated with breast cancer risk (P<0.05). In stage II, one SNP rs8752 was replicated at P<0.05. This SNP is located in the 3’ untranslated region (UTR) of the 15-hydroxyprostaglandin dehydrogenase (*HPGD*) gene at 4q34-35, a miR-485-5p binding site. Compared with the GG genotype, the combined GA+AA genotypes has a significantly higher risk of breast cancer (OR = 1.18; 95% CI: 1.06-1.31, P = 0.002). Specifically, this SNP was associated with estrogen receptor (ER) positive breast cancer (P = 0.0007), but not with ER negative breast cancer (P = 0.23), though p for heterogeneity not significant.

**Conclusion:**

Through a systematic case-control study of microRNA binding site SNPs, we identified a new breast cancer risk variant rs8752 in *HPGD* in Chinese women. Further studies are warranted to investigate the underling mechanism for this association.

## Introduction

Breast cancer is the most common female malignancy worldwide, and its incidence has been increasing during the past several decades in both developing and developed countries [1]. It is widely accepted that environmental and genetic factors contribute to the development of breast cancer. Despite environmental factors play an important role in breast cancer, the individual’s risk of breast cancer was determined by the genetic susceptibility. Numerous investigations have suggested that microRNAs are essential for various biological processes and diseases, including tumorigenesis [2], [3], [4], [5], [6], [7]. MicroRNA inhibits gene translation by binding to the 3’ UTRs of target mRNAs. In recent years, many studies have revealed that SNPs or mutations within microRNA binding sites may affect cancer susceptibility by disrupting miRNA-mRNA interaction and mRNA expression [8], [9], [10], [11]. Several bioinformatic methods have been introduced to predict candidate SNPs located in microRNA target sites, including “Patrocles”, and “PolymiRTS” [12], [13], [14], [15], [16]. Some case-control studies have been performed to investigate the association between SNPs in microRNA binding sites and breast cancer risk [17], [18], [19], [20], [21], [22]. Wang *et al.* found that a miRNA binding site SNP in the 3'-UTR region of the IL23R gene may be associated with the risk of breast cancer and contribute to the early development of breast cancer in Chinese women [23]. Teo *et al.* first reported the association between DNA repair gene *PARP1* miRNA-binding site SNP rs8679 and breast cancer risk [24]. Zheng *et al.* reported that the presence of SNPs at the miR-124 binding site may be a marker for breast cancer risk and prognosis [25]. Kontorovich *et al.* found that the heterozygotes carriers of SNP rs11169571 had an approximately 2 fold increased risk for developing breast cancer, whereas heterozygotes of the rs895819 SNP had an approximately 50% reduced risk for developing breast cancer [26]. Saetrom *et al.* suggested that allele-specific regulation of *BMPR1B* by miR-125b explains the observed disease risk [27]. Brendle et al. showed that the A allele of the *ITGB4* SNP rs743554 was associated with the negative hormone receptor status and bad breast cancer-specific survival, especially in women with more aggressive tumors [28]. However, most of them are candidate gene studies including only a few SNPs, which could not represent the whole situation of these SNPs in breast cancer etiology.

In this study, high-throughput SNP genotyping was used in a large case-control study including genome-wide microRNA binding site SNPs. The results may provide new insights into the cause of breast cancer, and new molecular markers for breast cancer diagnosis.

## Materials and Methods

### Ethics statement

The Ethics Committee of Tianjin Medical University Cancer Institute and Hospital approved the study protocol, and all patients and controls gave written informed consent before participating in the study.

### Study subjects

A total of 5869 individuals (2744 breast cancer cases and 3125 controls) were involved in this study. The cases were collected from Tianjin Medical University Cancer Hospital between January 1, 2006 and December 31, 2008 who were newly diagnosed and histologically confirmed breast cancer patients. At the same time, controls were enrolled from the nearby community, who were genetically unrelated to the patients and were frequency matched to the patients by age (±3 years). Our study comprised two stages, in stage I, we randomly selected 1349 patients and 1572 cancer-free female controls for SNP screening. In stage II, to validate the findings from stage I, the validation set of 1395 cases and 1553 controls were genotyped. Participants were interviewed by trained investigator using a systematic questionnaire about their demographic characteristics, personal habits, family history, occupational exposure history, eating habits, physical exercise, and reproductive factors. For the cases, clinical information on tumor features and disease severity, including morphology, tumor size, lymph node metastasis, organ metastasis, tumor stage, and status of estrogen receptor (ER) and progesterone receptor (PR) were also collected. Each participant provided 10 ml venous blood. The study was approved by the Institutional Review Board of Tianjin Medical University; informed consent was obtained from all patients.

### SNP selection

The “Patrocles” online database (http://www.patrocles.org/) was used to select genome-wide micro-RNA target SNPs. Among all the 5035 SNPs in microRNA binding site SNPs that the database provided, 1742 SNPs had been confirmed. SNPs that satisfy the following criteria were considered for inclusion: (1) SNPs were located in a microRNA-seed region binding site, and the seed region was defined according to the "7-mirs" criteria [12]. (2) SNPs have reported population frequency data in Chinese (htpp://www.ncbi.nlm.nih.gov/snp/), and SNPs with minor genotype frequency ≥0.05 were included. In this way, a total of 192 microRNA target SNPs were included in our study, the detailed information for these SNPs can be seen in Table S1 in File S1.

### Genomic DNA samples

The whole blood samples from each participants were collected and stored in Vacutainer tubes (BD Franklin Lakes, NJ) containing anticoagulant of EDTA. Total genomic DNA was extracted from the whole blood using QIAGEN DNA Extraction Kit (QIAGEN Inc.). The extracted DNA was stored at -20°C in TE buffer.

### SNP genotyping

In stage I, SNP genotyping was conducted using Illumina Golden Gate SNP Genotyping Arrays according to the manufacturer’s instructions. Only plates with a consistent high call rate in the initial calling were used. If the call rate was <80%, we repeat the experiment. In stage II, genotyping were performed using the TaqMan platform in 384-well plates and read with the Sequence Detection Software on an ABI Prism 7900 instrument according to the manufacturer’s instructions (Applied Biosystems, Foster City, CA). Primers and probes were supplied by Applied Biosystems, the PCR conditions used were as follows: 50°C for 2 minutes, 95°C for 10 minutes, and 60°C for 1 minute for 40 cycles. After 2 rounds of genotyping, the success rate for genotyping was 99%, and 5% of the samples were selected for replication, the results were 100% concordant.

### Statistical analysis

A χ^2^ test was used to evaluate the differences in the distributions of major demographic variables and environmental risk factors, as well as the genotypes of selected SNPs between the breast cancer cases and controls. The Hardy-Weinberg equilibrium was determined by a χ^2^ goodness of fit test in controls. Unconditional logistic regression was used to examine the association between the SNPs and breast cancer risk by estimating the odds ratios (ORs) and 95% confident intervals (CIs), with and without adjustments for age, smoking status, menopause status, oral contraception use, history of benign breast diseases, and family history of cancer. All statistical tests were two-sided, and a P value of 0.05 was considered significant, correction for multiple comparisons was not performed. We used the SAS software version 9.0 (SAS Institute) for all statistical analyses.

## Results

The demographic characteristics of the 2744 breast cancer cases and 3125 cancer-free controls (Combined stage I and II) were presented in Table 1. Age was matched between cases and controls (P = 0.447). The differences in smoking status (P <0.001), oral contraceptive usage (P <0.001), menopause (P <0.001), history of benign breast diseases (P <0.001), and family history of cancer (P <0.001) were statistically significant between cases and controls. These characteristics were comparable between samples from stage I and samples from stage II (Table S2 in File S1).

**Table 1:** Characteristics of breast cancer cases and cancer-free controls (Stage I and II).

	No. (%)			
Variables	Cases (n = 2744)	Controls (n = 3125)	*P* value a	OR (95% CI)
Age				
≦55	1839 (67.0)	2065 (66.1)	0.447	
>55	905 (33.0)	1060 (33.9)		
Menopause b				
No	1299 (47.3)	1133 (36.7)	<0.001	1.00
Yes	1448 (52.7)	1950 (63.3)		0.65(0.58, 0.72)
Oral contraception use b				
Never	2115 (82.4)	2583 (86.5)	<0.001	1.00
Ever	452 (17.6)	403 (13.5)		1.37 (1.18, 1.59)
Smoking status b				
Never	2311 (88.0)	2907 (95.0)	<0.001	1.00
Ever	316 (12.0)	153 (5.0)		2.60(2.13, 3.18)
History of benign breast disease b				
Never	1995 (73.6)	2634 (86.3)	<0.001	1.00
Ever	716 (26.4)	417 (13.7)		2.27(1.98, 2.59)
Family history of cancer b ^,^ c				
No	1908 (69.4)	2475 (80.4)	<0.001	1.00
Yes	843 (30.6)	603 (19.6)		1.81 (1.61, 2.05)

a Two-sided *X* ^2^ test, *P*< 0.05 was considered statistically significant.

b Due to missing values, the number of patients was <2744 and that of controls was <3125 in the test set

c First- and second-degree relatives with history of cancer.

In stage I, among the 192 candidate SNPs, 15 SNPs showed a significant association with breast cancer risk at P<0.05. The associated SNPs were rs17264436 in *PBRM1* gene, rs1044145 in *YOD1* gene, rs1325774 in *CLDN10* gene, rs2654981 in *IGF1R* gene, rs7359387 in *NFAT5* gene, rs1047499 in *SPTBN1* gene, rs1180342 in *BMP8A* gene, rs1056796 in *MLANA* gene, rs8410 in *PREPL* gene, rs1130741 in *MPI* gene, rs8752 in *HPGD* gene, rs2466551 in *NFYB* gene, rs1059111 in *NEFL* gene, rs2530310 in *CNTNAP2* gene, and rs698761 in *PREPL* gene (Table 2).

**Table 2:** Logistic regression analysis of associations between polymorphisms in microRNA targets and the risk of breast cancer (Stage I and II).

	Stage I						Stage II				
	No. (%)						No. (%)				
Polymorphisms	Cases (n = 1349)	Controls (n = 1572)	*P* value	OR (95%CI)	Adjusted OR (95%CI) a		Cases (n = 1395)	Controls (n = 1553)	*P* value	OR (95%CI)	Adjusted OR (95%CI) a
Rs17264436 (T/A)											
TT	449 (33.31)	423 (26.91)	0.0007	1.00	1.00		408 (29.46)	454 (29.54)	0.91	1.00	1.00
TA	622 (46.14)	810 (51.53)		0.72 (0.61, 0.86)	0.72 (0.59, 0.86)		710 (51.26)	778 (50.62)		1.03 (0.83, 1.27)	1.04 (0.85, 1.31)
AA	277 (20.55)	339 (21.56)		0.77 (0.63, 0.95)	0.76 (0.60, 0.95)		267 (19.28)	305 (19.84)		0.99 (0.83, 1.17)	1.01 (0.82, 1.21)
Rs1044145 (A/G)											
AA	811 (60.12)	958 (60.94)	0.0015	1.00	1.00		783 (56.53)	896 (58.30)	0.61	1.00	1.00
AG	485 (35.95)	509 (32.38)		1.13 (0.96, 1.32)	1.20 (1.01, 1.43)		530 (38.27)	567 (36.89)		1.04 (0.74, 1.47)	1.04 (0.75, 1.46)
GG	53 (3.93)	105 (6.68)		0.60 (0.42, 0.84)	0.59 (0.41, 0.86)		72 (5.20)	74 (4.81)		1.11 (0.79, 1.56)	1.15 (0.76, 1.58)
Rs1325774 (A/C)											
AA	1036 (76.85)	1215 (77.34)	0.0016	1.00	1.00		1067 (76.82)	1188 (76.99)	0.082	1.00	1.00
AC	283 (20.99)	346 (22.02)		0.96 (0.80, 1.15)	1.00 (0.82, 1.21)		311 (22.39)	329 (21.32)		1.05 (0.87, 1.25)	1.04 (0.88, 1.22)
CC	29 (2.15)	10 (0.64)		3.40 (1.65, 7.00)	3.90 (1.75, 8.68)		11 (0.79)	26 (1.69)		0.47 (0.22, 0.96)	0.53 (0.26, 1.03)
Rs2654981 (G/C)											
GG	479 (35.53)	472 (30.08)	0.0046	1.00	1.00		480 (34.61)	532 (34.57)	0.98	1.00	1.00
GC	640 (47.48)	786 (50.10)		0.80 (0.68, 0.95)	0.80 (0.66, 0.95)		658 (47.44)	735 (47.76)		0.99 (0.84, 1.16)	0.96 (0.83, 1.19)
CC	229 (16.99)	311 (19.82)		0.73 (0.59, 0.90)	0.75 (0.59, 0.94)		249 (17.95)	272 (17.67)		1.01 (0.82, 1.25)	1.05 (0.88, 1.36)
Rs7359387 (A/C)											
AA	551 (40.91)	651 (41.41)	0.0062	1.00	1.00		559 (40.27)	607 (39.39)	0.52	1.00	1.00
AC	637 (47.29)	677 (43.07)		1.11 (0.95, 1.30)	1.10 (0.93, 1.31)		639 (46.04)	739 (47.96)		0.95 (0.75, 1.19)	0.94 (0.70, 1.26)
CC	159 (11.80)	244 (15.52)		0.77 (0.61, 0.97)	0.79 (0.62, 1.02)		190 (13.69)	195 (12.65)		1.07 (0.91, 1.25)	1.02 (0.76, 1.38)
Rs1047499 (A/G)^b^											
AA	389 (28.90)	489 (31.13)	0.0089	1.00	1.00						
AG	658 (48.89)	804 (51.18)		1.03 (0.87, 1.22)	0.97 (0.81, 1.17)						
GG	299 (22.21)	278 (17.70)		1.35 (1.10, 1.67)	1.30 (1.03, 1.64)						
Rs1180342 (A/C)											
AA	407 (30.24)	423 (26.98)	0.0157	1.00	1.00		426 (30.69)	421 (28.10)	0.22	1.00	1.00
AC	702 (52.15)	808 (51.53)		0.90 (0.76, 1.07)	0.91 (0.75, 1.09)		662 (47.69)	759 (50.67)		0.93 (0.76, 1.15)	0.90 (0.75, 1.13)
CC	237 (17.61)	337 (21.49)		0.73 (0.59, 0.91)	0.74 (0.59, 0.94)		300 (21.61)	318 (21.23)		1.08 (0.90, 1.31)	1.10 (0.92, 1.32)
Rs1056796 (C/A)											
CC	349 (25.93)	448 (28.59)	0.0165	1.00	1.00		378 (27.25)	415 (26.93)	0.84	1.00	1.00
CA	702 (52.15)	734 (46.84)		1.23 (1.03, 1.46)	1.25 (1.04, 1.52)		665 (47.95)	755 (48.99)		0.97 (0.81, 1.14)	0.95 (0.77, 1.19)
AA	295 (21.92)	385 (24.57)		0.98 (0.80, 1.21)	0.96 (0.77, 1.21)		344 (24.80)	371 (24.08)		1.02 (0.83, 1.25)	1.00 (0.82, 1.22)
Rs8410 (G/A)											
GG	625 (46.43)	720 (45.83)	0.0170	1.00	1.00		609 (44.10)	694 (45.06)	0.83	1.00	1.00
GA	557 (41. 38)	706 (44.94)		0.91 (0.78, 1.06)	0.85 (0.72, 1.01)		610 (44.17)	674 (43.77)		1.04 (0.82, 1.32)	1.03 (0.79, 1.36)
AA	164 (12.18)	145 (9.23)		1.30 (1.02, 1.67)	1.28 (0.97, 1.68)		162 (11.73)	172 (11.17)		1.07 (0.84, 1.37)	1.10 (0.86, 1.39)
Rs1130741 (A/G)											
AA	802 (59.45)	927 (59.12)	0.0321	1.00	1.00		827 (59.63)	932 (60.44)	0.069	1.00	1.00
AG	463 (34.32)	575 (36.67)		0.93 (0.80, 1.09)	0.93 (0.78, 1.10)		483 (34.82)	552 (35.80)		0.99 (0.82, 1.13)	1.00 (0.83, 1.15)
GG	84 (6.23)	66 (4.21)		1.47 (1.05, 2.06)	1.56 (1.08, 2.26)		77 (5.55)	58 (3.76)		1.33 (1.05, 2.03)	1.28 (0.99, 2.05)
Rs8752 (G/A)											
GG	567 (42.09)	715 (45.51)	0.0378	1.00	1.00		578 (41.64)	716 (46.40)	0.018	1.00	1.00
GA	642 (47.66)	675 (42.97)		1.20 (1.03, 1.40)	1.22 (1.05, 1.44)		654 (47.12)	686 (44.46)		1.18 (1.01, 1.38)	1.19 (1.02, 1.40)
AA	138 (10.24)	181 (11.52)		0.96 (0.75, 1.23)	0.97 (0.76, 1.26)		156 (11.24)	141 (9.14)		1.37 (1.06, 1.77)	1.38 (1.08, 1.79)
Rs2466551 (A/C)											
AA	560 (41.57)	600 (38.17)	0.0418	1.00	1.00		543 (39.12)	579 (37.55)	0.58	1.00	1.00
AC	608 (45.14)	717 (45.61)		0.91 (0.78, 1.06)	0.94 (0.79, 1.12)		638 (45.97)	738 (47.86)		0.91 (0.79, 1.07)	0.90 (0.76, 1.08)
CC	179 (13.29)	255 (16.22)		0.75 (0.60, 0.94)	0.84 (0.65, 1.07)		207 (14.91)	225 (14.59)		0.98 (0.79, 1.22)	0.96 (0.77, 1.20)
Rs1059111 (T/A)											
TT	587 (43.64)	623 (39.66)	0.0419	1.00	1.00		608 (43.84)	653 (42.35)	0.62	1.00	1.00
TA	600 (44.61)	726 (46.21)		0.88 (0.75, 1.03)	0.91 (0.76, 1.07)		603 (43.48)	678 (43.97)		0.94 (0.75, 1.18)	0.95 (0.79, 1.22)
AA	158 (11.75)	222 (14.13)		0.76 (0.60, 0.95)	0.73 (0.57, 0.94)		176 (12.69)	211 (13.68)		0.90 (0.71,1.13)	0.86 (0.68, 1.17)
Rs2530310 (A/G)											
AA	554 (41.13)	672 (42.83)	0.0465	1.00	1.00		622 (44.78)	675 (43.75)	0.49	1.00	1.00
AG	619 (45.95)	740 (47.16)		1.02 (0.87, 1.19)	1.03 (0.87, 1.23)		586 (42.19)	682 (44.20)		0.95 (0.75, 1.19)	0.96 (0.78, 1.18)
GG	174 (12.92)	157 (10.01)		1.34 (1.05, 1.72)	1.42 (1.09, 1.85)		181 (13.03)	186 (12.05)		1.07 (0.92, 1.25)	0.94 (0.70, .128)
Rs698761 (G/A)											
GG	615 (45.62)	716 (45.63)	0.0478	1.00	1.00		597 (42.98)	676 (43.81)	0.82	1.00	1.00
AG	564 (41.84)	699 (44.55)		0.94 (0.81, 1.10)	0.89 (0.75, 1.05)		626 (45.07)	693 (44.91)		1.06 (0.83, 1.34)	1.18 (0.16, 1.61)
AA	169 (12.54)	154 (9.82)		1.28 (1.00, 1.63)	1.22 (0.94, 1.60)		166 (11.95)	174 (11.28)		1.08 (0.85, 1.37)	1.21 (0.88, 1.66)

a ORs were adjusted for age, smoking status, menopause status, oral contraception use, history of benign breast diseases, and family history of cancer.

b due to failure in probe design, rs1047499 (A/G) was not genotyped in stage II.

In stage II, among the 15 SNPs identified from stage I, the SNP rs8752 in *HPGD* gene (the duplex structure between miR-485-5p and HPGD was shown in Figure S1 in File S1) was significantly associated with breast cancer risk in an independent replication set (P = 0.018). When data from stage I and stage II were combined, compared with rs8752 GG genotype, GA and AA genotypes were associated with a higher risk of breast cancer (OR =  1.18; 95% CI: 1.06–1.31, P = 0.002). Furthermore, we assessed these associations between rs8752 and breast cancer risk according to ER and PR status. The association was significant for ER positive breast cancer (OR = 1.24; 95% CI: 1.10–1.40, P = 0.0007), but not significant for ER negative breast cancer (OR = 1.09; 95% CI: 0.95–1.26, P = 0.23), though p for heterogeneity not significant. The association between rs8752 and breast cancer risk was similar for PR positive and PR negative breast cancer (Table 3).

**Table 3:** Logistic regression analysis of associations between rs8752 and the risk of breast cancer (Stage I and II).

	No. (%)				
Polymorphisms	Cases (n = 2735)	Controls (n = 3114)	*P* value	OR (95%CI)	Adjusted OR (95%CI) a
Rs8752 (G/A) (Stage I)					
GG	567 (42.09)	715 (45.51)	0.038	1.00	1.00
GA	642 (47.66)	675 (42.97)		1.20 (1.03, 1.40)	1.22 (1.05, 1.44)
AA	138 (10.24)	181 (11.52)		0.96 (0.75, 1.23)	0.97 (0.76, 1.26)
GA+AA	780 (57.91)	856(54.49)	0.050	1.15 (0.99, 1.33)	1.17 (1.01, 1.35)
Rs8752 (G/A) (Stage II)					
GG	578 (41.64)	716 (46.40)	0.018	1.00	1.00
GA	654 (47.12)	686 (44.46)		1.18 (1.01, 1.38)	1.19 (1.02, 1.40)
AA	156 (11.24)	141 (9.14)		1.37 (1.06, 1.77)	1.38 (1.08, 1.79)
GA+AA	810 (58.36)	827 (53.60)	0.012	1.21 (1.05. 1.40)	1.22 (1.06, 1.41)
Rs8752 (G/A) (Combined)					
GG	1145 (41.86)	1431 (45.95)	0.006	1.00	1.00
GA	1296 (47.39)	1361 (43.71)		1.19 (1.07, 1.33)	1.20 (1.08, 1.34)
AA	294 (10.75)	322 (10.34)		1.14 (0.96, 1.36)	1.15 (0.97, 1.38)
GA+AA	1590 (58.14)	1683 (54.05)	0.002	1.18 (1.06, 1.31)	1.19 (1.07, 1.32)
Rs8752 (G/A) (ER-)					
GG	443 (43.77)	1431 (45.95)	0.23	1.00	1.00
GA+AA	569 (56.23)	1683 (54.05)		1.09 (0.95, 1.26)	1.10 (0.96, 1.28)
Rs8752 (G/A) (ER+)					
GG	631 (40.71)	1431 (45.95)	0.0007	1.00	1.00
GA+AA	919 (59.29)	1683 (54.05)		1.24 (1.10, 1.40)	1.26 (1.08, 1.45)
Rs8752 (G/A) (PR-)					
GG	491 (41.54)	1431 (45.95)	0.009	1.00	1.00
GA+AA	691 (58.46)	1683 (54.05)		1.20 (1.05, 1.37)	1.17 (1.03, 1.34)
Rs8752 (G/A) (PR+)					
GG	583 (42.22)	1431 (45.95)	0.02	1.00	1.00
GA+AA	798 (57.78)	1683 (54.05)		1.16 (1.02, 1.32)	1.19 (1.05, 1.36)

a ORs were adjusted for age, smoking status, menopause status, oral contraception use, history of benign breast diseases, and family history of cancer.

## Discussion

In this study, we performed a two-stage case-control study including 2744 cases and 3125 controls. Among the 192 SNPs genotyped, the SNP rs8752 (A allele) in *HPGD* gene was identified to be associated with an increased risk of breast cancer in both stages. This study provided a piece of evidence for a novel susceptibility variation for breast cancer on chromosome 4q34-35.

Our study covered three SNPs (*IGF1R* rs2654981, *NFAT5* rs7359387, *NELF* rs1059111) that were previously studied in the context of breast cancer risk. The *IGF1* receptor (*IGF1R*) overexpression has been associated with a number of hematological neoplasias and solid tumors including breast cancer [29]. Han-Sung Kang found that seven of the 51 *IGF1R* SNPs were in LD (linkage disequilibrium) and in one haplotype block, and were likely to be associated with breast cancer risk [30]. Sebastien Jauliac found that *NFAT5* were expressed in invasive human ductal breast carcinomas and participate in promoting carcinoma invasion using cell lines derived from human breast and colon carcinomas [31]. *NEFL* has been shown to act as a tumor suppressor in the carcinogenesis of breast [32], [33]. However, we found significant association between these three SNPs and breast cancer risk only in stage I. In stage II (the validation set),we did not find significant association.

The microRNA-related SNPs can generally be categorized into three groups, SNPs in microRNA sequences, SNPs in microRNA biogenesis pathway genes, and SNPs in microRNA target sites [34], [35], [36]. Up to now, SNPs in microRNA sequences and microRNA biogenesis pathway genes had been systematically studied and important findings were reported from these studies [37], [38]. However, for the association between microRNA binding site SNPs and breast cancer risk, most previous studies were based on candidate gene strategy. Results from these studies were not enough to represent the role of such SNPs in the etiology of cancer. In this sense, we conducted a systematic case–control study including genome-wide microRNA binding site SNPs.

The *HPGD* gene at chromosome 4q34-35 encodes a short-chain non-metalloenzyme alcohol dehydrogenase protein family. The encoded enzyme is responsible for the metabolism of prostaglandins, which function in a variety of physiologic and cellular processes, such as inflammation. HPGD is widely distributed in various mammalian tissues such as lung, breast, prostate, placenta and gut. Recent studies have shown a reduction of HPGD in some cancers, such as colorectal, breast, prostate, and lung [39], [40], [41], [42], [43]. Many studies have revealed that HPGD may have tumor-suppressive properties [44], [45]. Ido Wolf *at al.* reported that HPGD was an epigenetically silenced tumor suppressor gene in breast cancer and there was an association between HPGD expression and the ER pathway activity. Prostaglandin E2 (PGE2) is a major stimulator of expression of aromatase, thus leading to increased synthesis of estrogen within the breast [40]. PGE2 levels are regulated not only by its synthesis but also by its degradation. The key enzyme responsible for the biological inactivation of prostaglandins is NAD+-linked HPGD [41]. Our results add another dimension to the above findings that the A allele of *HPGD* had a positive association with breast cancer risk, and the association was ER status specific. SNP rs8752 (G/A) is located in the miR-485-5p binding site, and it is likely to disrupt the miR-485-5p/HPGD interaction. As shown in Figure S1 in File S1, the A allele cannot be targeted by miR-485-5p, which will result in the increase of HPGD protein expression, a possible underlying mechanism for the observed association with the risk of breast cancer.

Although, to the best of our knowledge, this is the largest systematic case-control study investigating the association between microRNA target SNPs and breast cancer risk. Our study has several limitations. First, we included only the SNPs with high frequency of variation, namely three genotypes with minor genotype frequency ≥0.05. This strategy will inevitably miss some low frequency SNPs that associated with breast cancer risk. Second, functional studies are critical to confirm the findings of association from this study, while such studies were not performed at this stage. Third, correction for multiple comparisons was not performed in this study, although our design with large sample size and replication set can ensure a high repeatability of our findings.

In summary, our findings suggested that common variants in the *HPGD* gene might be associated with breast cancer risk among Chinese women. Further large studies are warranted to confirm these findings and to examine the biological mechanisms for the association.

## Supporting Information

File S1 **Supporting file including Figure S1, Table S1, and Table S2. Table S1.** 192 microRNA binding site SNPs identified from “Patrocles” database. **Table S2.** Characteristics of breast cancer cases and cancer-free controls (Stage I and Stage II).**Figure S1.** The duplex structure of miR-485-5p and the 3’UTR of HPGD gene.
(DOCX)

## References

[1] Jemal A, Bray F, Center MM, Ferlay J, Ward E, et al (2011) Global cancer statistics. CA Cancer J Clin 61: 69–90. 21296855 10.3322/caac.20107

[2] Babashah S, Soleimani M (2011) The oncogenic and tumour suppressive roles of microRNAs in cancer and apoptosis. Eur J Cancer 47: 1127–1137. 21402473 10.1016/j.ejca.2011.02.008

[3] Fowler A, Thomson D, Giles K, Maleki S, Mreich E, et al (2011) miR-124a is frequently down-regulated in glioblastoma and is involved in migration and invasion. Eur J Cancer 47: 953–963. 21196113 10.1016/j.ejca.2010.11.026

[4] Yan LX, Huang XF, Shao Q, Huang MY, Deng L, et al (2008) MicroRNA miR-21 overexpression in human breast cancer is associated with advanced clinical stage, lymph node metastasis and patient poor prognosis. RNA 14: 2348–2360. 18812439 10.1261/rna.1034808 PMC2578865

[5] Yu Z, Wang C, Wang M, Li Z, Casimiro MC, et al (2008) A cyclin D1/microRNA 17/20 regulatory feedback loop in control of breast cancer cell proliferation. J Cell Biol 182: 509–517. 18695042 10.1083/jcb.200801079 PMC2500136

[6] Wu H, Zhu S, Mo YY (2009) Suppression of cell growth and invasion by miR-205 in breast cancer. Cell Res 19: 439–448. 19238171 10.1038/cr.2009.18 PMC2664859

[7] Winter J, Diederichs S (2011) MicroRNA biogenesis and cancer. Methods Mol Biol 676: 3–22. 20931386 10.1007/978-1-60761-863-8_1

[8] Chen K, Song F, Calin GA, Wei Q, Hao X, et al (2008) Polymorphisms in microRNA targets: a gold mine for molecular epidemiology. Carcinogenesis 29: 1306–1311. 18477647 10.1093/carcin/bgn116

[9] Landi D, Gemignani F, Naccarati A, Pardini B, Vodicka P, et al (2008) Polymorphisms within micro-RNA-binding sites and risk of sporadic colorectal cancer. Carcinogenesis 29: 579–584. 18192692 10.1093/carcin/bgm304

[10] Saetrom P, Biesinger J, Li SM, Smith D, Thomas LF, et al (2009) A risk variant in an miR-125b binding site in BMPR1B is associated with breast cancer pathogenesis. Cancer Res 69: 7459–7465. 19738052 10.1158/0008-5472.CAN-09-1201 PMC2747041

[11] Chin LJ, Ratner E, Leng S, Zhai R, Nallur S, et al (2008) A SNP in a let-7 microRNA complementary site in the KRAS 3' untranslated region increases non-small cell lung cancer risk. Cancer Res 68: 8535–8540. 18922928 10.1158/0008-5472.CAN-08-2129 PMC2672193

[12] Hiard S, Charlier C, Coppieters W, Georges M, Baurain D (2010) Patrocles: a database of polymorphic miRNA-mediated gene regulation in vertebrates. Nucleic Acids Res 38: D640–D651. 19906729 10.1093/nar/gkp926 PMC2808989

[13] Bhattacharya A, Ziebarth JD, Cui Y (2014) PolymiRTS Database 3.0: linking polymorphisms in microRNAs and their target sites with human diseases and biological pathways. Nucleic Acids Res 42: D86–D91. 24163105 10.1093/nar/gkt1028 PMC3965097

[14] Yu Z, Li Z, Jolicoeur N, Zhang L, Fortin Y, et al (2007) Aberrant allele frequencies of the SNPs located in microRNA target sites are potentially associated with human cancers. Nucleic Acids Res 35: 4535–4541. 17584784 10.1093/nar/gkm480 PMC1935019

[15] Saunders MA, Liang H, Li WH (2007) Human polymorphism at microRNAs and microRNA target sites. Proc Natl Acad Sci U S A 104: 3300–3305. 17360642 10.1073/pnas.0611347104 PMC1805605

[16] Landi D, Gemignani F, Barale R, Landi S (2008) A catalog of polymorphisms falling in microRNA-binding regions of cancer genes. DNA Cell Biol 27: 35–43. 17941804 10.1089/dna.2007.0650

[17] Zhang L, Liu Y, Song F, Zheng H, Hu L, et al (2011) Functional SNP in the microRNA-367 binding site in the 3'UTR of the calcium channel ryanodine receptor gene 3 (RYR3) affects breast cancer risk and calcification. Proc Natl Acad Sci U S A 108: 13653–13658. 21810988 10.1073/pnas.1103360108 PMC3158174

[18] Song F, Zheng H, Liu B, Wei S, Dai H, et al (2009) An miR-502-binding site single-nucleotide polymorphism in the 3'-untranslated region of the SET8 gene is associated with early age of breast cancer onset. Clin Cancer Res 15: 6292–6300. 19789321 10.1158/1078-0432.CCR-09-0826

[19] Chen J, Qin Z, Jiang Y, Wang Y, He Y, et al (2014) Genetic Variations in the Flanking Regions of miR-101-2 Are Associated with Increased Risk of Breast Cancer. PLoS One 9: e86319. 24475105 10.1371/journal.pone.0086319 PMC3901682

[20] Gilam A, Edry L, Mamluk-Morag E, Bar-Ilan D, Avivi C, et al (2013) Involvement of IGF-1R regulation by miR-515-5p modifies breast cancer risk among BRCA1 carriers. Breast Cancer Res Treat 138: 753–760. 23549953 10.1007/s10549-013-2502-5

[21] Jedlinski DJ, Gabrovska PN, Weinstein SR, Smith RA, Griffiths LR (2011) Single nucleotide polymorphism in hsa-mir-196a-2 and breast cancer risk: a case control study. Twin Res Hum Genet 14: 417–421. 21962133 10.1375/twin.14.5.417

[22] Nicoloso MS, Sun H, Spizzo R, Kim H, Wickramasinghe P, et al (2010) Single-nucleotide polymorphisms inside microRNA target sites influence tumor susceptibility. Cancer Res 70: 2789–2798. 20332227 10.1158/0008-5472.CAN-09-3541 PMC2853025

[23] Wang L, Liu W, Jiang W, Lin J, Jiang Y, et al (2012) A miRNA binding site single-nucleotide polymorphism in the 3'-UTR region of the IL23R gene is associated with breast cancer. PLoS One 7: e49823. 23239971 10.1371/journal.pone.0049823 PMC3519811

[24] Teo MT, Landi D, Taylor CF, Elliott F, Vaslin L, et al (2012) The role of microRNA-binding site polymorphisms in DNA repair genes as risk factors for bladder cancer and breast cancer and their impact on radiotherapy outcomes. Carcinogenesis 33: 581–586. 22166496 10.1093/carcin/bgr300 PMC3291859

[25] Zheng H, Song F, Zhang L, Yang D, Ji P, et al (2011) Genetic variants at the miR-124 binding site on the cytoskeleton-organizing IQGAP1 gene confer differential predisposition to breast cancer. Int J Oncol 38: 1153–1161. 21318219 10.3892/ijo.2011.940

[26] Kontorovich T, Levy A, Korostishevsky M, Nir U, Friedman E (2010) Single nucleotide polymorphisms in miRNA binding sites and miRNA genes as breast/ovarian cancer risk modifiers in Jewish high-risk women. Int J Cancer 127: 589–597. 19950226 10.1002/ijc.25065

[27] Saetrom P, Biesinger J, Li SM, Smith D, Thomas LF, et al (2009) A risk variant in an miR-125b binding site in BMPR1B is associated with breast cancer pathogenesis. Cancer Res 69: 7459–7465. 19738052 10.1158/0008-5472.CAN-09-1201 PMC2747041

[28] Brendle A, Lei H, Brandt A, Johansson R, Enquist K, et al (2008) Polymorphisms in predicted microRNA-binding sites in integrin genes and breast cancer: ITGB4 as prognostic marker. Carcinogenesis 29: 1394–1399. 18550570 10.1093/carcin/bgn126

[29] Kang HS, Ahn SH, Mishra SK, Hong KM, Lee ES, et al (2014) Association of polymorphisms and haplotypes in the insulin-like growth factor 1 receptor (IGF1R) gene with the risk of breast cancer in Korean women. PLoS One 9: e84532. 24392142 10.1371/journal.pone.0084532 PMC3879335

[30] Jauliac S, Lopez-Rodriguez C, Shaw LM, Brown LF, Rao A, et al (2002) The role of NFAT transcription factors in integrin-mediated carcinoma invasion. Nat Cell Biol 4: 540–544. 12080349 10.1038/ncb816

[31] Li XQ, Li L, Xiao CH, Feng YM (2012) NEFL mRNA expression level is a prognostic factor for early-stage breast cancer patients. PLoS One 7: e31146. 22319610 10.1371/journal.pone.0031146 PMC3271096

[32] Kang S, Kim B, Park SB, Jeong G, Kang HS, et al (2013) Stage-specific methylome screen identifies that NEFL is downregulated by promoter hypermethylation in breast cancer. Int J Oncol 43: 1659–1665. 24026393 10.3892/ijo.2013.2094

[33] Chen Z, Xu L, Ye X, Shen S, Li Z, et al (2013) Polymorphisms of microRNA sequences or binding sites and lung cancer: a meta-analysis and systematic review. PLoS One 8: e61008. 23613771 10.1371/journal.pone.0061008 PMC3628762

[34] Lin M, Gu J, Eng C, Ellis LM, Hildebrandt MA, et al (2012) Genetic polymorphisms in MicroRNA-related genes as predictors of clinical outcomes in colorectal adenocarcinoma patients. Clin Cancer Res 18: 3982–3991. 22661538 10.1158/1078-0432.CCR-11-2951 PMC4141857

[35] Slaby O, Bienertova-Vasku J, Svoboda M, Vyzula R (2012) Genetic polymorphisms and microRNAs: new direction in molecular epidemiology of solid cancer. J Cell Mol Med 16: 8–21. 21692980 10.1111/j.1582-4934.2011.01359.x PMC3823089

[36] Yang H, Dinney CP, Ye Y, Zhu Y, Grossman HB, et al (2008) Evaluation of genetic variants in microRNA-related genes and risk of bladder cancer. Cancer Res 68: 2530–2537. 18381463 10.1158/0008-5472.CAN-07-5991

[37] Lin J, Horikawa Y, Tamboli P, Clague J, Wood CG, et al (2010) Genetic variations in microRNA-related genes are associated with survival and recurrence in patients with renal cell carcinoma. Carcinogenesis 31: 1805–1812. 20732906 10.1093/carcin/bgq168 PMC2981457

[38] Ryu YM, Myung SJ, Park YS, Yang DH, Song HJ, et al (2013) Inhibition of 15-hydroxyprostaglandin dehydrogenase by Helicobacter pylori in human gastric carcinogenesis. Cancer Prev Res (Phila) 6: 349–359. 23430757 10.1158/1940-6207.CAPR-12-0389 PMC3796116

[39] Wolf I, O'Kelly J, Rubinek T, Tong M, Nguyen A, et al (2006) 15-hydroxyprostaglandin dehydrogenase is a tumor suppressor of human breast cancer. Cancer Res 66: 7818–7823. 16885386 10.1158/0008-5472.CAN-05-4368

[40] Ding Y, Tong M, Liu S, Moscow JA, Tai HH (2005) NAD+-linked 15-hydroxyprostaglandin dehydrogenase (15-PGDH) behaves as a tumor suppressor in lung cancer. Carcinogenesis 26: 65–72. 15358636 10.1093/carcin/bgh277

[41] Backlund MG, Mann JR, Holla VR, Buchanan FG, Tai HH, et al (2005) 15-Hydroxyprostaglandin dehydrogenase is down-regulated in colorectal cancer. J Biol Chem 280: 3217–3223. 15542609 10.1074/jbc.M411221200 PMC1847633

[42] Tong M, Tai HH (2004) Synergistic induction of the nicotinamide adenine dinucleotide-linked 15-hydroxyprostaglandin dehydrogenase by an androgen and interleukin-6 or forskolin in human prostate cancer cells. Endocrinology 145: 2141–2147. 14749354 10.1210/en.2003-1229

[43] Huang G, Eisenberg R, Yan M, Monti S, Lawrence E, et al (2008) 15-Hydroxyprostaglandin dehydrogenase is a target of hepatocyte nuclear factor 3beta and a tumor suppressor in lung cancer. Cancer Res 68: 5040–5048. 18593902 10.1158/0008-5472.CAN-07-6575 PMC2762106

[44] Pham H, Eibl G, Vincenti R, Chong B, Tai HH, et al (2008) 15-Hydroxyprostaglandin dehydrogenase suppresses K-RasV12-dependent tumor formation in Nu/Nu mice. Mol Carcinog 47: 466–477. 18058808 10.1002/mc.20404

[45] Li M, Xie J, Cheng L, Chang B, Wang Y, et al (2008) Suppression of invasive properties of colorectal carcinoma SW480 cells by 15-hydroxyprostaglandin dehydrogenase gene. Cancer Invest 26: 905–912. 19034772 10.1080/07357900802146154

